# Enhanced vesicular stomatitis virus (VSVΔ51) targeting of head and neck cancer in combination with radiation therapy or ZD6126 vascular disrupting agent

**DOI:** 10.1186/1475-2867-12-27

**Published:** 2012-06-15

**Authors:** Nehad M Alajez, Joseph D Mocanu, Tiffany Krushel, John C Bell, Fei-Fei Liu

**Affiliations:** 1 Stem Cell Unit, Department of Anatomy, College of Medicine, King Saud University, Riyadh, Saudi Arabia; 2 Ontario Cancer Institute, Toronto, ON, Canada; 3 Department of Medical Biophysics, University of Toronto, Toronto, ON, Canada; 4 Centre for Cancer Therapeutics, Ottawa Health Research Institute, Ottawa, ON, Canada; 5 Department of Biochemistry, Microbiology and Immunology, University of Ottawa, Ottawa, ON, Canada; 6 Department of Radiation Oncology, University Health Network, Toronto, ON, Canada; 7 Department of Radiation Oncology, University of Toronto, Toronto, ON, Canada; 8 Department of Radiation Oncology, Princess Margaret Hospital/Ontario Cancer Institute, 610 University Avenue, Toronto, ON, Canada, M5G 2 M9

**Keywords:** Oncolytic virus, HNSCC, Radiotherapy, Vascular disrupting

## Abstract

**Background:**

Head and neck squamous cell carcinoma (HNSCC) is the 5th most common cancer worldwide. Locally advanced HNSCC are treated with either radiation or chemo-radiotherapy, but still associated with high mortality rate, underscoring the need to develop novel therapies. Oncolytic viruses have been garnering increasing interest as anti-cancer agents due to their preferential killing of transformed cells. In this study, we evaluated the therapeutic potential of mutant vesicular stomatitis virus (VSVΔ51) against the human hypopharyngeal FaDu tumour model *in vitro* and *in vivo*.

**Results:**

Our data demonstrated high toxicity of the virus against FaDu cells *in vitro*, which was associated with induction of apoptosis. *In vivo*, systemic injection of 1 × 10^9^ pfu had minimal effect on tumour growth; however, when combined with two doses of ionizing radiation (IR; 5 Gy each) or a single injection of the vascular disrupting agent (ZD6126), the virus exhibited profound suppression of tumour growth, which translated to a prolonged survival in the treated mice. Concordantly, VSVΔ51 combined with ZD6126 led to a significant increase in viral replication in these tumours.

**Conclusions:**

Our data suggest that the combinations of VSVΔ51 with either IR or ZD6126 are potentially novel therapeutic opportunities for HNSCC.

## Background

Head and neck squamous cell carcinoma (HNSCC) is the most common cancer type in the head and neck region, accounting for the 5th most common cancer worldwide [1]. Locally advanced diseases are treated with either radiation or chemo-radiotherapy, but are still associated with >50% mortality rate [1,2], underscoring the need to develop novel therapeutic strategies. Oncolytic viruses have recently garnered increasing interest as anti-cancer agents due to their preferential killing of transformed cells (reviewed in [3,4]). Among these, the mutant variant of vesicular stomatitis virus (VSV) has been evaluated in different tumour models, demonstrating promising results as either single agent or in combination with other treatment modalities [5-7]. We had previously demonstrated the exquisite sensitivity of EBV-positive nasopharyngeal carcinoma (NPC) to a mutant VSV (VSVΔ51), which has a single amino acid deletion in the VSV M protein, rendering lethality to cancer cells, whilst sparing normal cells [8]. Building upon this observation, herein we evaluated the efficacy of VSVΔ51 against the human FaDu hypopharyngeal squamous cell carcinoma model either as a single agent, or combined with radiation therapy (RT) or the vascular disrupting agent ZD6126. ZD6126 is a colchicine prodrug derivative that is metabolized *in vivo* to yield ZD6126 phenol, which then selectively binds to the colchicine-binding site of tubulin; this disrupts the microtubule structure, largely responsible for the structure and morphology of dividing and immature vascular endothelial cells [9]. Our data demonstrated enhanced efficacy of VSVΔ51 when combined with either RT or ZD6126 *in vivo*, thereby supporting the potential clinical utility of these combination strategies for head and neck cancer.

## Results

### VSVΔ51 is lethal to FaDu cells *in vitro*

**Figure 1:**
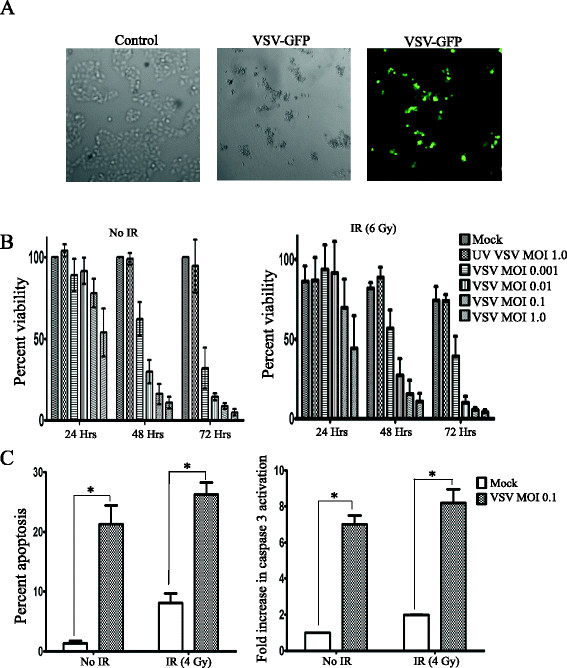
**VSV**Δ**51 is lethal to FaDu cells  *in vitro. * ** (**A**) FaDu Cells were visualized at 72 hrs post-infection with VSVΔ51 (0.1 pfu/cell) using light (middle) or fluorescence microscope (right) and compared to un-infected normal FaDu cells (left). (**B**) MTS assay for FaDu cell viability at 24, 48, and 72 hrs post-infection with the indicated doses of VSVΔ51 alone (left) or combined with 6 Gy IR (right) delivered just before infection. Data are presented as mean ± SD from representative experiments, n = 3. (**C**) Percent apoptosis (left panel) and fold increase in caspase 3 activity (right panel) in FaDu cells at 72 hrs post infection with 0.1 pfu/cell VSVΔ51. Data are presented as mean ± SE; n = 3 from two independent experiments.*p ≤ 0.05.

To evaluate sensitivity of HNSCC cells to VSVΔ51, FaDu cells were infected with VSVΔ51 at 0.1 pfu/cell. Figure 1A demonstrated significant toxicity of VSVΔ51 against FaDu cell at 72 hrs post infection (pi). Using the MTS cell viability assay, VSVΔ51 exhibited both dose-and time-dependent toxicity against FaDu cells (Figure 1B, left panel), which did not change significantly with the addition of 6 Gy ionizing radiation (IR, Figure 1B, right panel). Using cell cycle analysis, a significant proportion of cells underwent apoptotic cell death at 72 hrs pi with 0.01 pfu/cell (Figure 1C, left panel). Combining VSVΔ51 with 4 Gy IR led to a slight increase in apoptosis (26% *vs*. 22%). Similarly, induction of caspase 3 activation was observed in VSVΔ51 infected cells, thus confirming apoptosis as a mode of cell death in infected cells (Figure 1C, right panel).

### Efficacy of VSVΔ51 combined with radiation therapy (RT) or ZD6126 in established FaDu xenograft model

**Figure 2:**
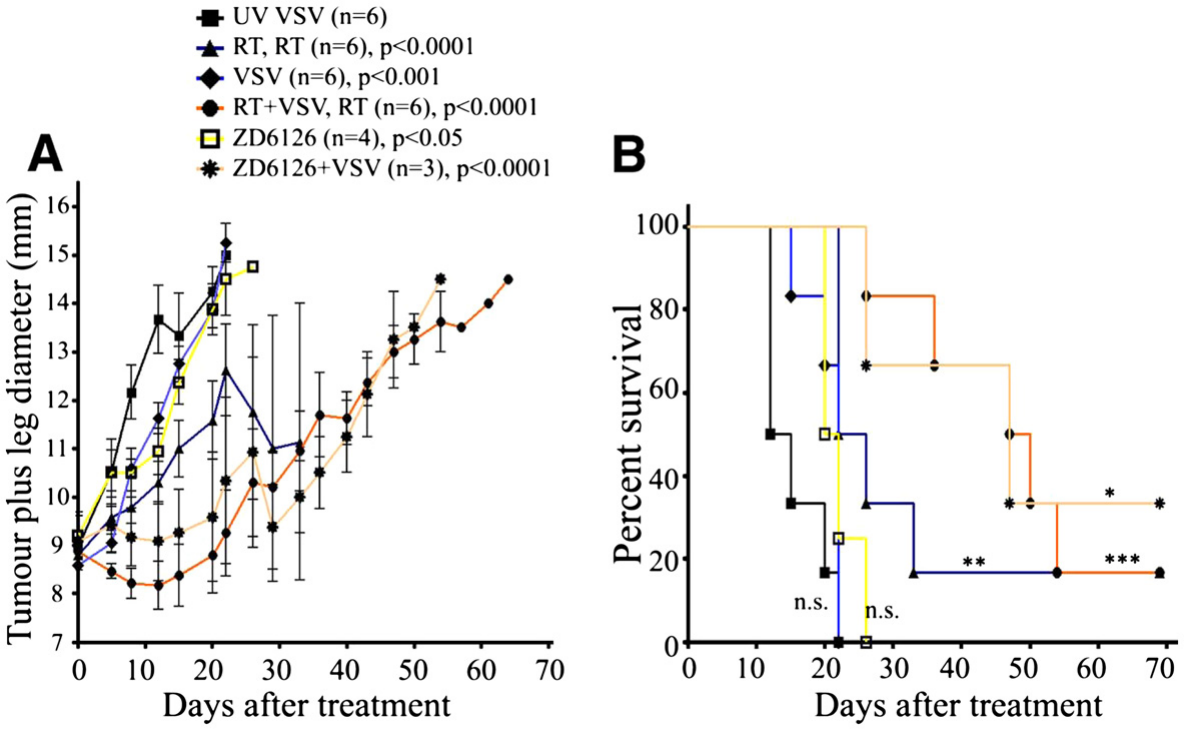
**Efficacy of VSVΔ51 combined with RT or ZD6126 in the FaDu xenograft model.** (**A**) FaDu xenograft tumours were established im in the left leg of CD-1 nude mice to allow for local RT delivery. Mice were then randomized to six groups: a) UV-inactivated VSVΔ51 (1 × 10^9^ pfu i.v.); (b) local tumour RTx2 (5 Gy), 3 days apart; (c) VSVΔ51 (1 × 10^9^ pfu i.v.); (d) RT (5 Gy each) plus VSVΔ51 (1 × 10^9^ pfu i.v.) simultaneously, then 2^nd^ RT (5 Gy) delivered 3 days later; (e) ZD6126 alone (5 mg i.v. in 200 μl); and (f) ZD6126 (5 mg) just preceding 1 × 10^9^ VSVΔ51 i.v. Data are presented as mean leg plus tumour diameter ± S.E. (**B**) Mice in panel (A) were monitored for survival for up to 70 days. All p-values were calculated using the Two-Way ANOVA analysis in comparison with the UV-inactivated VSV control group; survival comparisons were conducted using the log-rank test. n.s., not significant, *p≤0.05,** p≤0.005, ***p≤0.0005.

**Figure 3:**
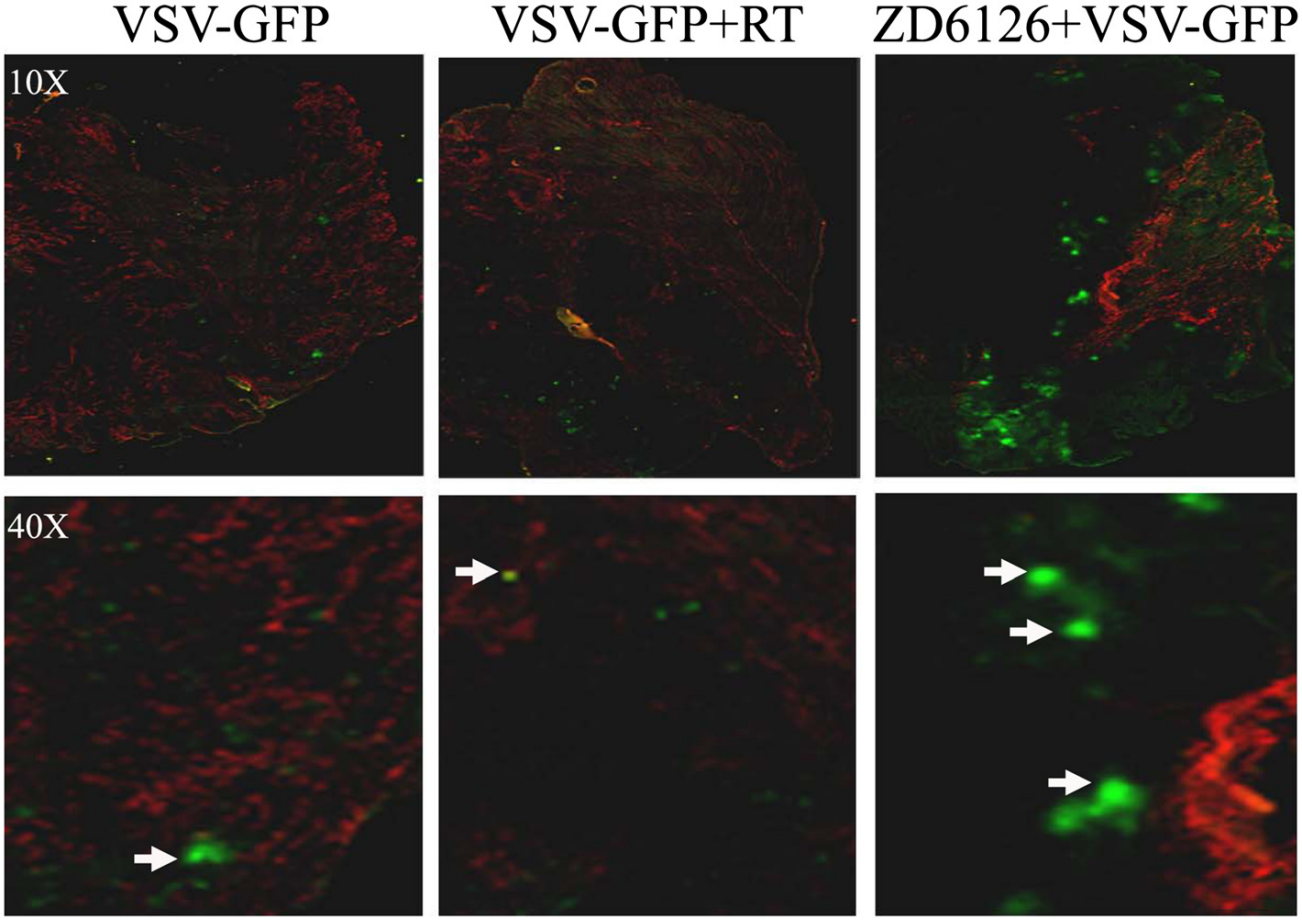
**Representative examples of VSVΔ51 tumour microdistribution in FaDu xenograft tumours.** Viral distribution (GFP) was assessed at 24 h post infection in relation to active vasculature (Hoechst, red). The merged field consisted of superimposed VSV, and Hoechst; the close-up images are high-resolution representations of GFP distribution in relation to local blood vessels.

To determine whether the observed *in vitro* toxicity of VSVΔ51 against FaDu cell could be recapitulated *in vivo*, FaDu cells were implanted intramuscularly (im) into the left leg of CD-1 nude mice. Once the leg plus tumour diameter reached ~9 mm, the mice were randomized to: a) UV-inactivated VSVΔ51; (b) local tumour RTx2 (5 Gy each), 3 days apart; (c) VSVΔ51 alone; (d) RT (5 Gy) plus VSVΔ51 simultaneously; then 2^nd^ RT (5 Gy) 3 days later; (e) ZD6126 alone; and (f) ZD6126 just preceding VSVΔ51 injection. VSVΔ51 alone had modest effects, whereas two doses of 5 Gy RT led to a significant reduction in tumour growth (Figure 2A). When combined with RT, VSVΔ51 was able to significantly further decrease tumour growth compared to RT alone. Interestingly, the vascular disrupting agent ZD6126 had minimal effect on tumour growth when administered alone, but when combined with a single dose of VSVΔ51, a significant inhibition of tumour growth was observed compared to either ZD6126 or VSVΔ51 alone (Figure 2A). Although the combination of VSVΔ51 with either RT or ZD6126 was not sufficient to completely eradicate the tumor, these combination led to the longest survival of tumour-bearing mice in that the median survival increased from ~22 (for the VSV alone group) to 48 and 47 days, respectively (Figure 2B). While no obvious increase in viral replication was observed in the RT plus VSVΔ51 group, images of tumour sections removed from treated mice revealed a significant increase in VSVΔ51 replication in the ZD6126 plus VSVΔ51-treated group, suggesting that the observed anti-tumour efficacy is likely attributable to enhanced viral deposition and replication in this group of mice (Figure 3).

## Discussion

In recent years, oncolytic viruses have emerged as promising anti-cancer agents due to their demonstrated efficacy in a number of preclinical models [10-13], exerting a myriad of anti-tumour mechanisms ranging from direct tumour cell lysis, induction of apoptosis, to targeting of the tumour vasculature [8,14,15]. Despite these promising pre-clinical data, clinical trials utilizing oncolytic viruses have demonstrated limited success; accentuating the need for improvement. To that end, several studies have administered oncolytic viruses in conjunction with standard or targeted therapies, demonstrating enhanced anti-tumour efficacy [16,17]. Our group has previously reported that increased tumour interstitial fluid pressure (IFP) could hinder therapeutic efficacy of adenoviral gene therapy *in vivo*[18]. As a follow-up, we also observed that the vascular disrupting agent ZD6126 could enhance bio-distribution of anti-sense oligonucleotides in xenografttumours, mediated *via* reducing tumour IFP [19]. These two observations were hence combined, to evaluate whether anti-tumour efficacy of VSVΔ51 could be improved with the addition of ZD6126. It was gratifying to observe that the combination of VSVΔ51 with ZD6126 was indeed effective in suppressing tumour growth, to a similar extent as that observed when VSVΔ51 was delivered in conjunction with local tumour RT (Figure 2A and B). Furthermore, enhanced viral presence was observed in the tumours treated with VSVΔ51 plus ZD6126 (Figure 3). The mechanisms could relate to both increased viral delivery consequent to tumour vasculature disruption leading to increased leakiness, plus reduced tumour IFP, both resulting in increased deposition and replication of the oncolytic virus.

When VSVΔ51 was combined with local tumour RT, enhanced viral replication was not observed, although effective anti-tumour activity was still apparent (Figures 2 &3). The extent of neutrophil infiltration was no different between the tumours treated with VSVΔ51 alone, or when combined with RT (data not shown), indicating that innate immunity cannot explain these results of the combination therapy. Hence, the mechanism of the additional tumour suppressive effect of VSVΔ51 combined with RT is likely an additive interaction between the cytotoxicity of the viral oncolytic therapy, plus the direct tumoricidal effects of ionizing radiation.

## Conclusions

Enhanced efficacy of VSVΔ51 in an HNSCC tumour model is documented when combined with either local tumour RT, or with the use of a vascular disrupting agent ZD2612 *in vivo.* These combinational regimens have disparate mechanisms of anti-tumour efficacy, and have the potential as novel therapeutic strategies in the management of HNSCC.

## Methods

### Ethics statement

All animal experiments were conducted in accordance with the guidelines of the Animal Care Committee, University Health Network, Toronto, Canada.

### Cell lines and reagents

FaDu human hypopharyngeal carcinoma cell line was purchased from the American Type Culture Collection (ATCC, Manassas, VA), and was authenticated at the Centre for Applied Genomics (the Hospital for Sick Children, Toronto, Canada) using the AmpF/STR Identifiler PCR Amplification Kit (Applied BiosystemsInc, Foster City, CA). Cells were cultured in in MEM-F15 supplemented with 10% fetal bovine serum (FBS), 100 mg/L penicillin, and 100 mg/L streptomycin (RPMI-10) at 37°C, 5% CO_2._ 293-F Cells were purchased from invitrogen (Invitrogen, Carlsbad, CA).

### Virus production and cell infection

VSVΔ51 carrying GFP (referred to as VSVΔ51 in this paper) was propagated in 293-F cells as previously described [8]. Briefly, 293-F cells were cultured in 293 SFM II medium (Invitrogen, Carlsbad, CA) in 500 mL spin-culture flasks, then were infected with VSVΔ51-GFP at MOI 0.01 in the presence of 1.8 mM CaCl_2_. Supernatant was collected 16–18 hours later, filtered through a 0.22 μm filter, then collected by centrifugation at 18,500 g for 2.5 hrs in a Sorvall RC5 centrifuge. UV inactivation was performed by exposing the virus for 30 min at 10 cm distance under UV light in a class II biosafety cabinet. For cell infection, 10,000 FaDu cells were seeded in 100 μL of MEM-F15 plus 10% FBS in a 96-well plate. Twenty-four hrs later, cells were exposed to 0 or 6 Gy IR, medium was removed, and VSVΔ51 (MOI = 1.0-0.001) was added in 20 μL α-MEM medium. The plates were kept at 37°C for 60 min to allow virus attachment prior to the addition of 80 μl of normal growth medium (MEM-F15 plus 10% FBS).

### Cell viability and measurement of apoptosis

Cell viability was assessed using the MTS assay as previously described [20], and according to the manufacturer-recommended protocol (Promega, Madison, WI). Briefly, 20 μL of the MTS reagent was added to each well in a 96-well plate at the indicated time points, and absorbance was read at λ_492_. In order to measure the fraction of cells in the subG0/G1 phase of cell cycle, FaDu cells were infected with VSVΔ51 at MOI 0.1; at indicated time points, cells were harvested, and then washed twice in FACS buffer (PBS/0.5% BSA). Cells were re-suspended in 1 mL of FACS buffer, then 3 mL of ice-cold 70% ethanol was added to fix the cells for 1 hr on ice. Cells were washed once, before re-suspending in 500 μL of FACS buffer supplemented with 40 μg/mL RNAseA (Sigma) and 50 μg/mL PI. Cells were incubated at room temperature for 30 min in the dark before being analyzed in the BD FACScalibur using FL-2A and FL-2 W channels. CaspGlow kit (Biovision, Mountain view, CA) was used to measure caspase 3 activity in virally-infected FaDu cells. Briefly, infected cells were re-suspended in 300 μL of RPMI-10 followed by the addition of 1 μL of FITC-labeled DEVD-FMK reagent for 45 min at 37°C to measure activated caspase 3. Following 45 mins’ incubation, cells were washed, re-suspended in buffer, then analyzed in BD FACScalibur using FL-1 channel.

### Animal experiments

Five to six week old CD-1 nude male mice were purchased from Charles River Laboratories (Montreal, Canada); all experiments were conducted in accordance with the guidelines of the Animal Care Committee, University Health Network. For therapeutic experiments, 3 × 10^6^ FaDu cells were injected intramuscular (im) into the left leg. When tumour plus leg diameter reached 8.5-9.5 mm, treatment was initiated. Mice were treated as indicated by injecting 1 × 10^9^ pfu VSVΔ51 in 100 μL PBS intravenously (iv), *via* the tail vein. For local radiation treatments, mice were immobilized in a Lucite box, and the tumor-bearing leg was exposed to 225 kVp (13 mA) at a dose rate of 3.37 Gy/min using an X-ray irradiator C (X-RAD 225; Precision X-ray)*.* ZD6126 (AstraZeneca) treatment was delivered as previously described [18]. Briefly, ZD6126 was dissolved in 10% sodium carbonate and 90% PBS (pH 7.4, 25 mg/mL). A single 200 μL injection of ZD6126 solution was injected i.p. into FaDu–bearing nude mice. For the combined treatment, mice were treated with ZD6126 and then immediately injected with 1 × 10^9^ pfu VSVΔ51 in 100 μL PBS intravenously (iv), *via* the tail vein. Tumor growth was monitored by measuring tumor plus leg diameter as described before [21].

### Microdistribution studies

FaDu xenograft tumours were established and treated as described above. At 24 hrs post-VSV injection, Hoechst 33342 (600 μg in 100 μL PBS) was injected iv for visualization of the active vasculature [22]; the mice were sacrificed 1 min later. Tumours were immediately excised, frozen in Optimal Cutting Temperature (OCT) compound (Bayer Corporation, USA), then stored at −80°C. Serial sections (5 μm thickness) were cut through each tumour at three levels, 500 μm apart. The slides were scanned using fluorescence microscopy to visualize GFP-expression and Hoechst 33342 perfusion. All staining and cryosectioning procedures were performed by Pathology Research Program Services, University Health Network (Toronto, Canada).

### Microscopy

Slides were imaged at 10X magnification using an Olympus BX50 (Olympus, Japan) tiling fluorescence stereomicroscope (FITC/GFP: λ_Ex_ = 482 nm, λ_Em_ = 535 nm, 500 ms exposure; Hoechst 33342: λ_Ex_ = 360 nm, λ_Em_ = 460 nm, 100 ms exposure). Images were captured using ImagePro 5.1 (Media Cybernetics, USA). Fluorescence microscopy images were imported into Adobe Photoshop and merged into a composite image, where the red, and green channels corresponded to Hoechst 33342 (active vasculature), and GFP (VSV infection), respectively.

### Statistical analysis

Statistical analysis and graphing were performed using Microsoft Excel 2010, and Graphpad Prism 5.0 software.

## Competing interests

There are no competing interests.

## Authors’ contributions

NMA designed and did the experiments and wrote the manuscript; JDM helped with sample processing and microscopy, TK helped with proliferation and apoptosis experiments, JCB provided VSVΔ51 virus, and FFL conceived the study and obtained funding.All authors read and approved the final manuscript.
